# Fentanyl Exposure Is Associated with Adverse Respiratory Outcomes in Ventilated Preterm Neonates: A Retrospective Matched Cohort Study

**DOI:** 10.3390/jcm15166457

**Published:** 2026-08-20

**Authors:** Maria Di Chiara, Gianluigi Laccetta, Maria Chiara De Nardo, Lucia Dito, Gianluca Terrin

**Affiliations:** Department of Maternal-Infantile and Urological Sciences, Sapienza University of Rome, Policlinico Umberto I, Viale del Policlinico 155, 00161 Rome, Italy; gianluigi.laccetta@uniroma1.it (G.L.); mariachiara.denardo@uniroma1.it (M.C.D.N.); gianluca.terrin@uniroma1.it (G.T.)

**Keywords:** bronchopulmonary dysplasia, fentanyl, mechanical ventilation, neonatal sedation, preterm infant

## Abstract

**Background/Objectives:** Fentanyl is widely used in ventilated preterm neonates, but respiratory safety evidence is limited. We assessed the association between fentanyl exposure during mechanical ventilation and respiratory outcomes in preterm neonates <34 weeks and explored a candidate exposure-duration threshold, defined as the duration of continuous fentanyl infusion beyond which the risk of adverse respiratory outcomes rises sharply. **Methods:** Single-centre retrospective cohort study at Policlinico Umberto I, Sapienza University, Rome (2015–2022). Fentanyl-exposed neonates were 1:2 matched to unexposed neonates on gestational age and birth-weight z-score. Primary outcome: bronchopulmonary dysplasia at 36 weeks postmenstrual age (multivariable logistic regression); secondary outcome: duration of invasive ventilation. A composite outcome (bronchopulmonary dysplasia or invasive ventilation ≥ 7 days) was used for threshold analysis with restricted cubic splines, maximally selected Wald χ^2^, and segmented logistic regression. **Results:** Of 315 eligible neonates, 50 fentanyl-exposed neonates were matched with 92 unexposed neonates (*n* = 142). Fentanyl exposure was associated, after adjustment for gestational age and fetal distress, with bronchopulmonary dysplasia (adjusted odds ratio 5.49, 95% CI 1.72–17.54) and longer invasive ventilation (adjusted exp(β) 2.25, 95% CI 1.54–3.29). Three complementary threshold methods were consistent with an exploratory signal around 3 days of continuous infusion, beyond which composite-outcome odds rose, although the breakpoint estimate was imprecise. **Conclusions:** Fentanyl exposure during mechanical ventilation was associated, after adjustment for gestational age and fetal distress, with bronchopulmonary dysplasia and longer invasive ventilation in preterm neonates <34 weeks. A possible signal around 3 days of continuous infusion is proposed as a hypothesis-generating stewardship prompt, not as a decision rule.

## 1. Introduction

Preterm neonates, in this study defined as infants born before 34 completed weeks of gestation, admitted to neonatal intensive care units are exposed to numerous painful and stressful procedures during their hospitalization, particularly when invasive mechanical ventilation is required [1,2]. Fentanyl, a synthetic opioid with rapid onset, is the most used analgesic and sedative agent in this population [3,4]. Its administration during mechanical ventilation has become a common clinical practice, with reported exposure rates exceeding 40% among extremely preterm infants [5].

Despite its widespread use, the evidence supporting the analgesic efficacy of fentanyl in ventilated preterm neonates remains limited and uncertain. The only multicenter randomized controlled trial of continuous fentanyl infusion in this population demonstrated a reduction in acute procedural pain scores during the first three days of treatment but no effect on prolonged pain associated with mechanical ventilation, alongside an increased proportion of neonates still ventilated at one week of life [6].

Available evidence on the safety and efficacy of fentanyl in ventilated preterm neonates is conflicting and inconclusive. The most recent Cochrane systematic review, including 23 trials and over 2000 neonates, concluded that opioids probably have little or no effect on the duration of mechanical ventilation or on neonatal mortality, with low to moderate certainty of evidence [7]. The most recent fentanyl-specific systematic review and meta-analysis, restricted to four trials and 268 preterm neonates, did not detect a significant effect on bronchopulmonary dysplasia, on duration of mechanical ventilation, or on mortality, with very low certainty of evidence [8]. Alongside this uncertain efficacy, growing observational evidence raises concerns about the safety profile of fentanyl in preterm neonates. Opioid exposure during mechanical ventilation has been associated with an increased risk of bronchopulmonary dysplasia (BPD), with prolonged duration of ventilation and longer hospital stay, and with higher rates of major neonatal morbidities [5,9,10]; a propensity score-matched cohort from the National Neonatal Research Database of England and Wales has further documented a dose-dependent association between opioid exposure and preterm brain injury [11]. BPD, the most common chronic respiratory morbidity of prematurity, was defined as the need for supplemental oxygen at 36 weeks of postmenstrual age, according to the criteria proposed by Jobe and Bancalari, a definition adopted to ensure comparability with the reference literature.

However, this heterogeneous evidence has not yet been translated into practical guidance for the bedside neonatologist. Most observational studies pool fentanyl with morphine or other sedatives, are based on administrative or registry datasets that lack detailed clinical data, and capture exposure only within the first postnatal days rather than over the entire ventilation course; no study to date has explored an exposure-duration threshold for fentanyl. The present study was designed to address these gaps.

The aim of the present study was to evaluate the association between fentanyl exposure during mechanical ventilation and respiratory outcomes: BPD and duration of invasive mechanical ventilation (IMV). As a secondary objective, we performed a pre-specified threshold analysis to explore a candidate exposure-duration cutpoint.

## 2. Materials and Methods

### 2.1. Study Design and Population

We designed a retrospective cohort study with prospectively collected data, conducted at the Neonatal Intensive Care Unit (NICU) of Policlinico Umberto I Hospital, Sapienza University of Rome, Italy. We screened all preterm neonates with a gestational age (GA) below 34 weeks who were consecutively admitted to the NICU between January 2015 and December 2022 and who received any form of mechanical ventilation (invasive and/or non-invasive) during their stay. All included neonates had a documented Premature Infant Pain Profile-Revised (PIPP-R) [12] score above 12 during the ventilation course, indicating moderate-to-severe pain according to the criteria adopted in the only multicentre Italian fentanyl trial in this population and consistent with the Italian Society of Neonatology recommendations on neonatal pain assessment [6]. Pain was assessed prospectively at standardized intervals throughout the ventilation course using the PIPP-R scale; a documented score above 12 was required for inclusion in both groups and, because pain assessment precedes and informs any decision to provide analgesia, in fentanyl-exposed neonates the qualifying score was documented before the infusion was started. We excluded neonates with major congenital malformations, inborn errors of metabolism, congenital or acquired immunodeficiency, congenital infections, incomplete clinical data, absence of a documented PIPP-R assessment, and death or transfer to another hospital before 72 h of life. Three hundred and fifteen neonates met the eligibility criteria. According to fentanyl administration during mechanical ventilation, we defined as the exposed group the neonates exposed to fentanyl (*n* = 67) and as the unexposed group the neonates not exposed to fentanyl (*n* = 248).

To minimize confounding by indication, exposed and unexposed neonates were pair-matched on gestational age (caliper ±1 week) and on birth-weight z-score (caliper ±0.5 standard deviation) using a 1:2 greedy nearest-neighbor algorithm without replacement. Because the no-replacement rule combined with the prespecified calipers can leave an exposed neonate with fewer than two eligible unexposed neonates, the algorithm allowed a variable ratio: each exposed neonate could be paired with up to two unexposed neonates, but a 1:1 pairing was retained when only one unexposed neonate was available within the calipers. Fifty fentanyl-exposed neonates were successfully matched with 92 unexposed neonates, yielding an analytic cohort of 142 neonates. Seventeen fentanyl-exposed neonates, predominantly those with a gestational age between 22 and 26 weeks (a range in which fentanyl-exposed neonates outnumbered unexposed ventilated neonates, so that the 1:2 ratio applied without replacement frequently left no eligible unexposed neonate within the prespecified calipers), could not be matched within the prespecified calipers and were excluded from the analytic cohort. The sample size was determined by the number of consecutive eligible neonates during the study period; no formal a priori sample size calculation was performed. The onset of respiratory support was taken as the common clinical index time for both groups, as it defines eligibility, is documented for every neonate and necessarily precedes fentanyl exposure.

### 2.2. Exposure

The primary exposure was fentanyl administration during mechanical ventilation. According to the institutional NICU protocol, fentanyl was administered as a continuous intravenous infusion at a dose of 1 µg/kg/h. Fentanyl was the only agent used for continuous analgesia and sedation during mechanical ventilation in our unit. Exposure was operationalized both as a binary variable (any fentanyl administration during mechanical ventilation, yes vs. no) and as a continuous variable (duration of fentanyl exposure in days, computed from the recorded start and stop dates of the infusion). For the categorical exposure-response analysis, fentanyl exposure duration was further classified into tertiles of the exposed distribution (≤2 days, 3 to 5 days, >5 days), with the unexposed group as the reference category, yielding four ordered categories. Three exposed neonates whose start and stop dates fell on the same calendar day, corresponding to a continuous infusion shorter than 24 h, were assigned to the ≤2 days category to align the categorical variable with the binary exposure flag. Given the documented uncertainty regarding the balance between analgesic efficacy and respiratory safety of continuous fentanyl in this population, the decision to initiate fentanyl in eligible neonates with documented moderate-to-severe pain was made on a case-by-case basis at the discretion of the attending neonatologist.

### 2.3. Outcomes

The primary outcome was BPD, defined as the need for supplemental oxygen at 36 weeks of postmenstrual age, according to the criteria proposed by Jobe and Bancalari [13]. The secondary outcome was the duration of invasive mechanical ventilation, expressed in days. A composite respiratory outcome, defined as bronchopulmonary dysplasia at 36 weeks of postmenstrual age or invasive mechanical ventilation lasting at least 7 days, was prespecified for the threshold analysis of fentanyl exposure duration.

### 2.4. Data Collection

For each neonate, antenatal, perinatal and neonatal data were retrieved from the prospective NICU registry and from the clinical chart. Antenatal and obstetric variables included maternal age above 35 years, gestational diabetes, maternal thyroid disorders, antenatal infectious risk, intrauterine growth restriction, placental abruption, complete antenatal corticosteroid prophylaxis (defined as the administration of at least two doses) and multiple pregnancy. Perinatal and neonatal variables included gestational age (weeks), birth weight (grams), birth-weight z-score and small-for-gestational-age status, computed with reference to sex-specific Italian Neonatal Study (INeS) growth charts [14] sex, mode of delivery, fetal distress (defined as umbilical cord pH below 7.2 or 5 min Apgar score below 5) and respiratory distress syndrome requiring at least one dose of surfactant, used as a marker of disease severity.

### 2.5. Ethics

The study was conducted in compliance with the principles of the Declaration of Helsinki for medical research involving human subjects and was approved by the Ethics Committee of Policlinico Umberto I, Sapienza University of Rome (protocol no. 5089, 13 September 2018). Data were drawn from a prospectively maintained, ethics-approved NICU cohort whose protocol prespecified the collection of the analyzed variables (respiratory support, pharmacological therapy and morbidities of prematurity, including BPD). Written informed consent for the use of the child’s anonymized clinical data for clinical care and retrospective observational research was obtained from the parents or legally designated guardians of every neonate at admission; accordingly, a waiver of consent was not required. Individual-level data cannot be publicly shared owing to Italian privacy legislation (D.Lgs. 196/2003); de-identified data are available from the corresponding author upon reasonable request and after the appropriate institutional review.

### 2.6. Statistical Analysis

Analyses were performed in Python 3.11 using NumPy 2.4.4, SciPy 1.17.1. Fentanyl-exposed and unexposed neonates were 1:2 greedy nearest-neighbor matched without replacement on gestational age (±1 week) and birth-weight z-score (±0.5 SD). Continuous data: median (IQR), Mann–Whitney; categorical: *n*/*N* (%), χ^2^ or Fisher. Balance after matching was assessed with standardized mean differences (SMD), reported in Table 1 alongside *p*-values [15]. BPD at 36 weeks PMA (primary outcome) was analyzed by multivariable logistic regression and duration of invasive mechanical ventilation (secondary outcome) by ordinary least-squares regression on ln(duration + 1); both adjusted for gestational age and fetal distress, and additionally for RDS requiring surfactant in a sensitivity model and re-fitted in the surfactant-treated RDS subgroup. The association between fentanyl exposure duration and respiratory outcomes across four ordered categories was tested by Cochran–Armitage and Jonckheere–Terpstra. For a composite outcome (BPD or invasive ventilation ≥ 7 days), non-linearity was tested by restricted cubic splines [16], the optimal cutpoint by maximally selected Wald χ^2^ with Lausen–Schumacher correction [17], and the change-point by segmented logistic regression with 1000 bootstrap resamples. Two-sided *p* < 0.05 was considered significant.

## 3. Results

### 3.1. Participant Flow

**Figure 1:**
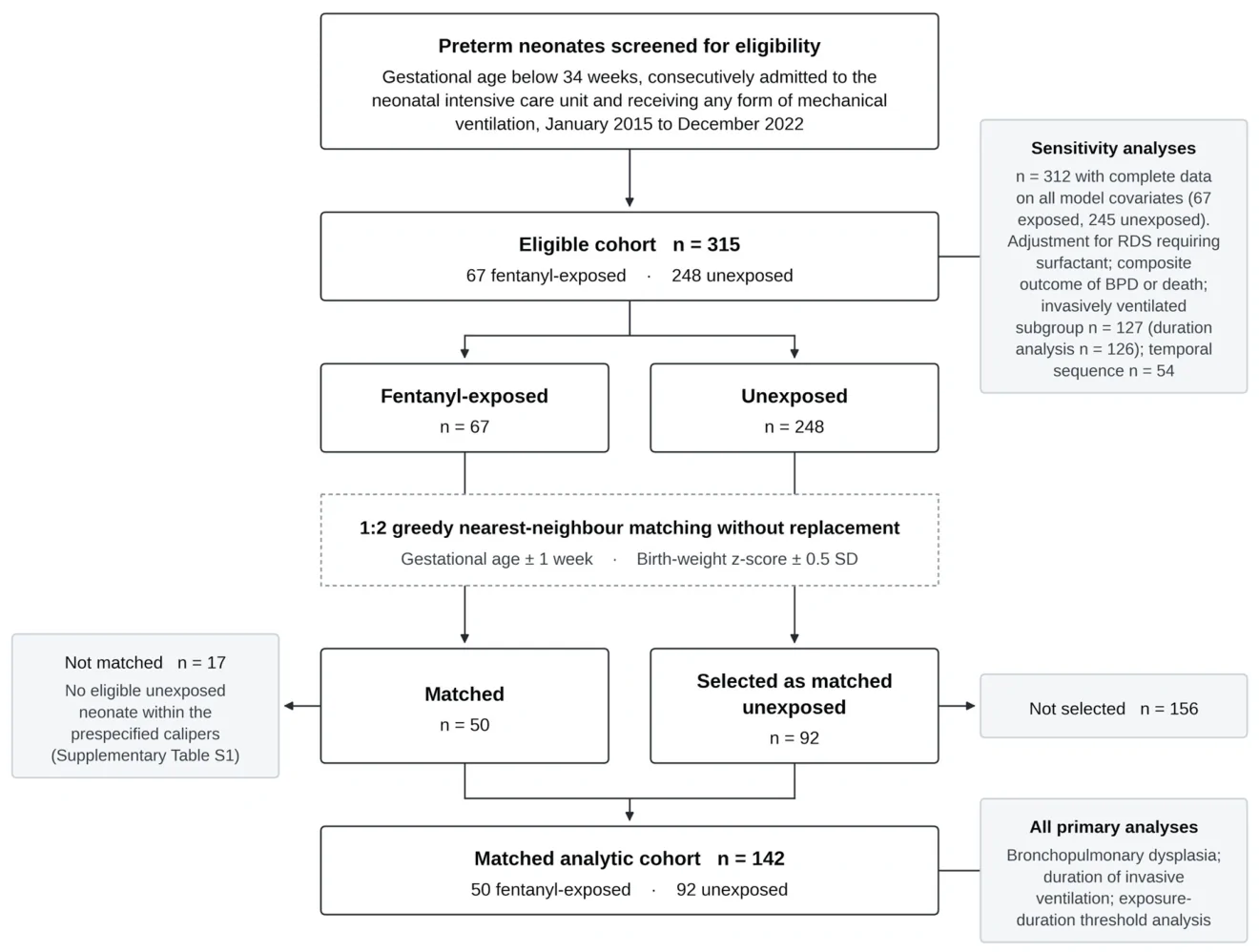
Participant flow. Preterm neonates below 34 weeks of gestation consecutively admitted to the neonatal intensive care unit of Policlinico Umberto I, Sapienza University of Rome, between January 2015 and December 2022 and receiving any form of mechanical ventilation were screened for eligibility. Neonates with major congenital malformations, inborn errors of metabolism, congenital or acquired immunodeficiency, congenital infections, incomplete clinical data, absence of a documented PIPP-R assessment, or death or transfer to another hospital before 72 h of life were not eligible. Shaded boxes indicate neonates not carried forward at each step and the samples used for each set of analyses. The box with a dashed border describes the matching procedure and does not represent a group of neonates. BPD, bronchopulmonary dysplasia; PIPP-R, Premature Infant Pain Profile-Revised; RDS, respiratory distress syndrome; SD, standard deviation.

Between January 2015 and December 2022, 315 preterm neonates with a gestational age below 34 weeks who received any form of mechanical ventilation during their stay in the neonatal intensive care unit of Policlinico Umberto I, Rome, were retrospectively identified and considered eligible for this cohort study. Of these, 67 had received fentanyl during the ventilation course (exposed) and 248 had not (unexposed). Complete data on both matching variables were available for 312 of these neonates (67 exposed and 245 unexposed), who constituted the population for the sensitivity analyses reported below. Exposed and unexposed neonates were matched 1:2 by gestational age (±1 week) and birth-weight z-score (±0.5 standard deviations), using a greedy nearest-neighbor algorithm without replacement. Fifty fentanyl-exposed neonates were successfully matched with 92 unexposed neonates, yielding an analytic cohort of 142 neonates. Seventeen fentanyl-exposed neonates (predominantly those with gestational age between 22 and 26 weeks, a range in which fentanyl-exposed neonates outnumbered unexposed ventilated neonates, so that the 1:2 ratio applied without replacement frequently left no eligible unexposed neonate within the prespecified calipers) could not be matched and were excluded from the analytic cohort. The participant flow, including the samples used for each set of analyses, is shown in Figure 1.

### 3.2. Baseline Characteristics

Baseline characteristics of the matched cohort are presented in Table 1. Matching closely balanced both stratification variables (birth-weight z-score, standardized mean difference −0.07; gestational age, −0.18, corresponding to a mean difference of 0.4 weeks, well within the prespecified caliper of ±1 week). Among characteristics not included in the matching algorithm, the absolute standardized mean difference exceeded 0.10 for respiratory distress syndrome requiring surfactant (1.06), fetal distress (0.49), multiple pregnancy (−0.32), male sex (−0.22), maternal thyroid disorders (0.19), maternal age above 35 years (−0.18), birth weight (−0.13) and complete antenatal steroid prophylaxis (−0.12). The two largest imbalances, and the only ones reaching statistical significance, were fetal distress (absolute risk difference between exposed and unexposed neonates 22.3%, 95% CI 6.1 to 38.4; *p* = 0.006) and respiratory distress syndrome requiring at least one dose of surfactant (absolute risk difference 44.5%, 95% CI 31.0 to 58.1; *p* < 0.001).

### 3.3. Unadjusted Respiratory Outcomes

Unadjusted respiratory outcomes are reported in Table 2. The odds of bronchopulmonary dysplasia were higher in fentanyl-exposed neonates (unadjusted odds ratio 6.11, 95% CI 2.03 to 18.38; *p* < 0.001). The duration of invasive mechanical ventilation was also longer in fentanyl-exposed neonates (Hodges-Lehmann median difference 4.0 days, 95% CI 2.0 to 5.0; *p* < 0.001).

### 3.4. Multivariable Analyses

**Figure 2:**
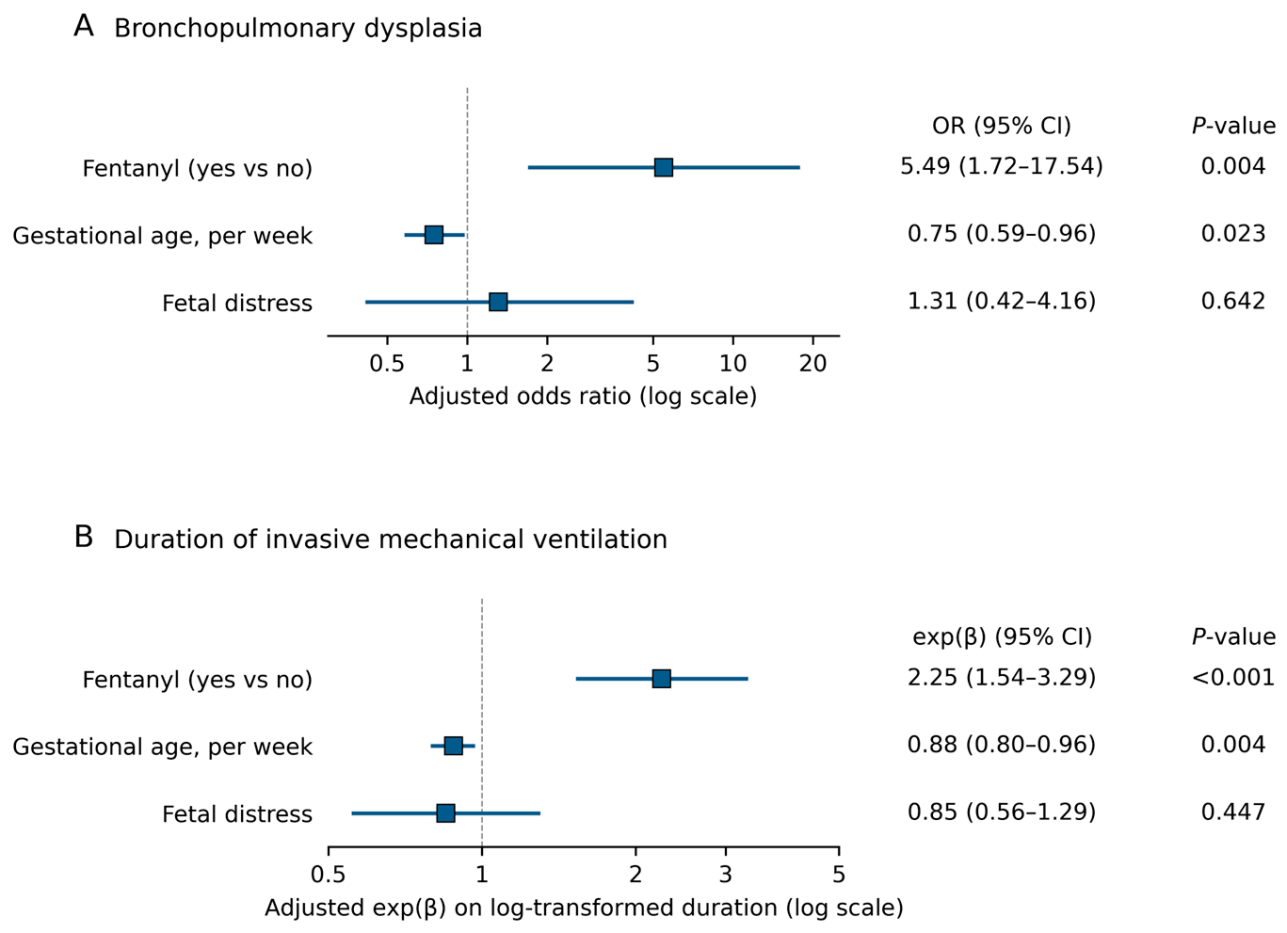
Multivariable adjusted associations between fentanyl exposure, gestational age, and fetal distress with respiratory outcomes in the matched cohort. (**A**) Logistic regression for bronchopulmonary dysplasia (BPD), defined as the need for supplemental oxygen at 36 weeks postmenstrual age (*n* = 142; BPD events = 18); effect estimates are adjusted odds ratios. (**B**) Linear regression on log-transformed duration of invasive mechanical ventilation, restricted to neonates with at least one day of invasive ventilation (*n* = 77); effect estimates are adjusted exp(β), interpretable as multiplicative factors on the duration of ventilation. Both models included fentanyl exposure (yes vs. no), gestational age (continuous, per week), and fetal distress (binary; umbilical cord pH below 7.2 or 5 min Apgar score below 5). Squares indicate point estimates and horizontal lines indicate 95% confidence intervals; the dashed vertical line marks the null effect (ratio = 1). The horizontal axis is on a logarithmic scale. CI confidence interval, OR odds ratio.

Multivariable regression results are reported in Figure 2. After adjustment for gestational age and fetal distress, fentanyl exposure remained associated with higher odds of bronchopulmonary dysplasia (adjusted odds ratio 5.49, 95% CI 1.72 to 17.54; *p* = 0.004) and with longer duration of invasive mechanical ventilation among the 77 neonates who received at least one day of invasive ventilation (adjusted exp(β) 2.25, 95% CI 1.54 to 3.29; *p* < 0.001).Within the matched cohort, further adjustment for RDS requiring surfactant attenuated but did not eliminate the association (adjusted OR 4.19, 95% CI 1.24 to 14.08, *p* = 0.021, versus 5.49 before adjustment; RDS: adjusted OR 2.77, 95% CI 0.54 to 14.25). Because only 18 bronchopulmonary dysplasia events were available for four covariates, the model was refitted with Firth’s penalized likelihood, which yielded a comparable estimate (adjusted OR 3.78, 95% CI 1.24 to 12.95). In a sensitivity analysis conducted in the eligible cohort of ventilated preterm neonates below 34 weeks with complete data on the matching variables (*n* = 312), further adjustment for RDS requiring surfactant, in addition to gestational age and fetal distress, attenuated but did not eliminate the association between fentanyl exposure and BPD (adjusted OR 4.74, 95% CI 1.57 to 14.32, *p* = 0.006, versus 6.46 before adjustment for RDS; RDS: adjusted OR 4.63, 95% CI 0.99 to 21.77), indicating that baseline lung disease contributes to but does not account for the association. In the same population, restricting to invasively ventilated neonates (*n* = 126), the duration of invasive ventilation remained longer in exposed than in unexposed neonates (median 6 versus 1 day; *p* < 0.001), with fentanyl initiated during ongoing invasive ventilation in 48 of 54 neonates (89%); invasive mechanical ventilation began on postnatal day 0 (IQR 0 to 0), fentanyl was initiated on day 1 (IQR 0 to 3), and invasive ventilation ended on day 6 (IQR 3 to 20). In a further sensitivity analysis restricted to the 127 invasively ventilated neonates, fentanyl exposure remained associated with bronchopulmonary dysplasia after adjustment for gestational age, fetal distress and RDS requiring surfactant (adjusted OR 3.68, 95% CI 1.19 to 11.43). Respiratory support began on postnatal day 0 in both groups (median 0, interquartile range 0 to 0 in exposed and unexposed neonates; *p* = 0.62), providing a common clinical index time; among exposed neonates fentanyl was started a median of 1 day (interquartile range 0 to 2) after the onset of invasive ventilation, and within 3 days in 85%.

### 3.5. Exposure-Response Analysis

**Figure 3:**
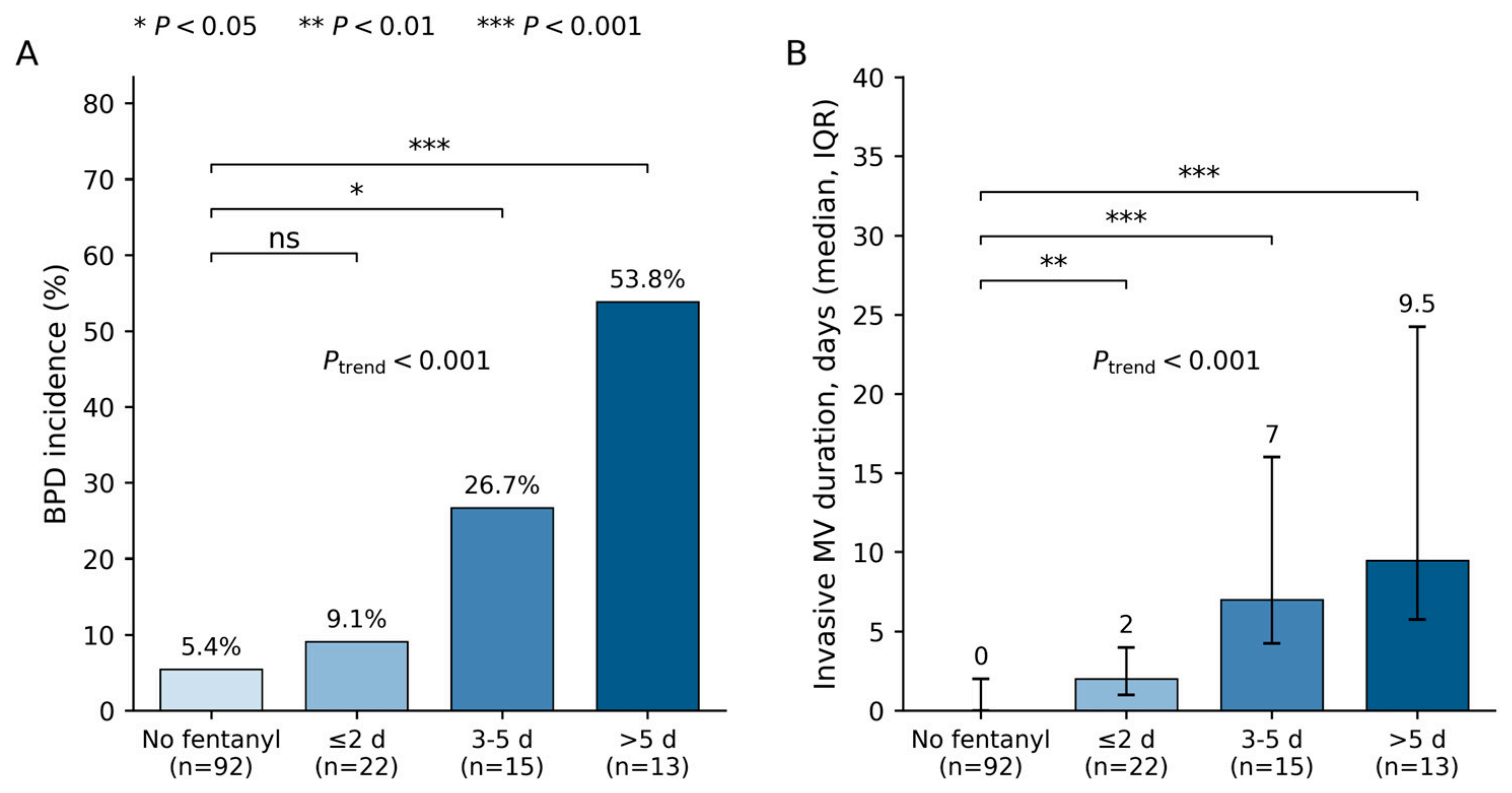
Association between fentanyl exposure duration and respiratory outcomes in the matched cohort. (**A**) Incidence of bronchopulmonary dysplasia (BPD), defined as oxygen requirement at 36 weeks postmenstrual age. (**B**) Duration of invasive mechanical ventilation (IMV), shown as median with interquartile range (error bars). The matched cohort comprised 142 ventilated preterm neonates (50 fentanyl-exposed, 92 unexposed) obtained by 1:2 greedy nearest-neighbor matching on gestational age (±1 week) and birth-weight z-score (±0.5 standard deviation), without replacement. Among fentanyl-exposed neonates, exposure duration was categorized in tertiles (≤2 days, 3–5 days, and >5 days); three exposed neonates with start–stop dates on the same calendar day (continuous infusion < 24 h) were assigned to the ≤2 days category to align with the binary exposure flag. *p* for trend across the four ordered categories was tested with the Cochran–Armitage test for BPD and the Jonckheere-Terpstra test for IMV duration. Brackets indicate pairwise comparisons against the no-fentanyl reference category, performed with Fisher’s exact test (BPD) and the Mann–Whitney U test (IMV duration). * *p* < 0.05; ** *p* < 0.01; *** *p* < 0.001; ns, not significant. BPD bronchopulmonary dysplasia, IQR interquartile range.

Exposure-response analyses are shown in Figure 3. Across the four ordered fentanyl exposure categories (no fentanyl, ≤2 days, 3–5 days, and >5 days), the trend was significant for both bronchopulmonary dysplasia (Cochran-Armitage z = 4.93, *p* < 0.001) and the duration of invasive mechanical ventilation (Jonckheere-Terpstra z = 6.49, *p* < 0.001).

### 3.6. Subgroup Analysis Stratified by RDS Requiring Surfactant

In a subgroup analysis stratified by respiratory distress syndrome requiring at least one dose of surfactant, fentanyl exposure was associated with both outcomes among the 84 neonates with RDS requiring surfactant: bronchopulmonary dysplasia (16 events; adjusted odds ratio 6.01, 95% CI 1.47 to 24.65; *p* = 0.013) and duration of invasive mechanical ventilation (adjusted exp(β) 2.17, 95% CI 1.39 to 3.41; *p* = 0.001). The corresponding models could not be reliably estimated in the 58 neonates without RDS requiring surfactant because of the small number of bronchopulmonary dysplasia events (*n* = 2) and of invasively ventilated neonates (*n* = 14).

### 3.7. Threshold Analysis

After adjustment for gestational age and fetal distress, the relationship between fentanyl exposure duration and the composite respiratory outcome was non-linear (restricted cubic spline test, χ^2^ = 8.98, *p* = 0.011). The maximally selected Wald statistic identified 3 days (72 h) as the optimal cutpoint (adjusted odds ratio 15.36, 95% CI 4.84 to 48.76; Lausen–Schumacher corrected *p* < 0.001), and segmented logistic regression placed the breakpoint at 3.5 days (bootstrap 95% CI 1.0 to 24.0). Cutpoint-specific adjusted odds ratios across candidate cutpoints from 1 to 8 days are reported in Table 3. Although these three approaches address distinct questions, the restricted cubic spline testing for departure from linearity and the maximally selected Wald statistic and segmented regression localizing the inflection point, they were consistent with a signal around 3 days of continuous exposure rather than identifying an identical point estimate; the wide bootstrap interval of the segmented-regression breakpoint is reported above to avoid over-precision.

## 4. Discussion

In this matched cohort study, designed to evaluate the association between fentanyl exposure during mechanical ventilation and respiratory outcomes in preterm neonates born below 34 weeks of gestation, we found that fentanyl exposure was associated, after adjustment for gestational age and fetal distress, with both a higher incidence of BPD at 36 weeks of postmenstrual age and a longer duration of IMV. The association was monotonic across four ordered categories of exposure duration, was reproduced in the pre-specified clinically most relevant subgroup of neonates with respiratory distress syndrome who had received at least one dose of surfactant and was consistent across three complementary threshold methods, which pointed to an exploratory signal around 3 days of continuous exposure.

Available systematic reviews and meta-analyses on the respiratory effects of fentanyl in ventilated preterm neonates have not detected significant associations. The most recent fentanyl-specific meta-analysis pooled four randomized trials and 268 preterm neonates and reported no significant effect on BPD, on duration of mechanical ventilation, or on mortality, with GRADE certainty rated as low to very low [8]. The broader Cochrane review on opioids during neonatal mechanical ventilation reached the same conclusion of probable little or no effect, although its pooled estimates are largely weighted by a single morphine trial and the fentanyl-specific contribution is too small to detect an effect of the magnitude we observed [7]. The most recent systematic review on the topic, performed by our group, reported that most cohort studies reviewed showed a prolonged duration of mechanical ventilation in opioid-exposed preterm neonates and explicitly recommended caution in the routine use of opioids during mechanical ventilation, particularly in extremely preterm infants [18].

The discordance between these null pooled estimates and the positive signal observed in real-world cohorts is biologically and methodologically consistent with the exposure-duration signal observed in the present study. The randomized trials feeding the available meta-analyses cap fentanyl exposure at a maximum of seven days by protocol, and the second largest fentanyl-specific randomized trial administered a mean exposure of approximately 75 h [6,19]. Although the multicenter trial by Ancora and colleagues was not powered for BPD, both the proportion of neonates still ventilated at one week of life and the durations of the first cycle of mechanical ventilation were significantly higher in the fentanyl arm, which is in full agreement with our findings on IMV duration; the smaller trial by Lago and colleagues similarly observed numerically higher rates of BPD and longer durations of ventilation in the fentanyl arm, all underpowered to reach significance but consistent in direction. The analgesic tolerance after the third day of continuous fentanyl reported by Ancora and colleagues themselves provides an independent pharmacological correlate of the same temporal landmark. We therefore raise the hypothesis that, with prolonged infusion, the cumulative respiratory cost of continuous fentanyl exposure may begin to exceed its analgesic benefit; this remains to be tested prospectively.

Among observational studies, the closest comparator is a recent retrospective propensity score-matched cohort of very preterm infants admitted to a Chinese tertiary neonatal intensive care unit, which used an exposure definition very close to ours (fentanyl and/or midazolam continuous infusion ≥24 h for non-procedural use) and reported an adjusted odds ratio for moderate-to-severe BPD or death superimposable on ours [9]. A nationwide propensity score-matched analysis from a Japanese administrative database reported higher BPD and longer length of intubation in opioid-exposed extremely preterm neonates, with the fentanyl-specific subgroup showing the same direction; however, that study captured opioid exposure only within the first two postnatal days, did not have access to drug dose, modality, or granular perinatal variables, and did not perform a dose–response or threshold analysis, limitations explicitly acknowledged by the authors [10]. Our matched cohort addresses each of these gaps by capturing fentanyl-specific exposure over the entire ventilation course through prospectively recorded start-stop dates and by examining the exposure-duration signal quantitatively. Two further large-scale observational studies on opioids during mechanical ventilation reported safety signals consistent in direction with our findings, although their primary outcomes (preterm brain injury and descriptive morbidity prevalence, respectively) differed from ours [5,11].

Several biologically plausible mechanisms support a role of prolonged fentanyl exposure on respiratory morbidity in this population, and these mechanisms have been described by the authors of the studies cited above. Pharmacological tolerance to the analgesic effect of continuous fentanyl is documented after the third day of treatment [6], which is biologically consistent with our threshold finding and predicts that any analgesic benefit beyond approximately 3 days of continuous infusion is at best uncertain. Prolonged opioid exposure is also associated with drug accumulation, with rapid tachyphylaxis, and with increased ventilatory pressure due to chest wall rigidity [5], each of which can contribute to a longer duration of IMV. Sustained sedation reduces spontaneous respiratory drive, prolongs the time on invasive ventilation, and thereby increases the cumulative exposure of the developing lung to volutrauma, oxidative stress, and inflammation, which are well-established drivers of BPD [10]. The non-linear shape of our exposure-response relationship, with a steep rise in the composite respiratory outcome above approximately 3 days of continuous exposure, is consistent with a tolerance-and-accumulation pattern in which the analgesic benefit plateaus while the ventilation-prolonging effect continues to accumulate.

From a clinical standpoint, the signal around 3 days may represent a hypothesis-generating stewardship prompt for the bedside neonatologist, to be confirmed in prospective studies. If confirmed, its potential utility would derive from being expressed in a unit already monitored at the cot-side (days of continuous infusion), corresponding to a cumulative dose of approximately 36 to 72 µg/kg. We do not interpret this finding as a threshold of safety in the toxicological sense, but as a stewardship signal: we emphasize that the segmented-regression breakpoint had a wide bootstrap interval and that the composite outcome incorporated invasive ventilation of at least 7 days, so that exposure duration and this component of the outcome are partly linked; the finding should therefore be read as hypothesis-generating. Whether continuous fentanyl infusion should be re-evaluated after approximately 3 days in the absence of a clear ongoing analgesic indication is a question for prospective evaluation, and would be consistent with the principle of selective and lowest-effective-dose use endorsed by the Cochrane review and consistent with the original observation already made by the only fentanyl-specific multicenter randomized trial [6,7]. Adequately powered prospective studies should test structured tapering protocols against the current pattern of open-ended continuous infusion.

Several strengths of the present study deserve mention. The exposure was specific to fentanyl, not pooled with morphine or with other sedative agents, which is the most common limitation of the available large-database studies. The analytic cohort was restricted to ventilated neonates, which provides a clinically homogeneous indication for sedation and limits the confounding by indication compared with unselected preterm cohorts. The matched design balanced the two strongest determinants of neonatal respiratory morbidity (gestational age and birth-weight z-score) by design, and the multivariable models additionally adjusted for fetal distress as the most relevant residual perinatal confounder. Because the institutional NICU protocol prescribes fentanyl as a continuous infusion at a standardized rate of 1 µg/kg/h, the duration of exposure is a faithful surrogate of the cumulative dose, with 3 days of exposure corresponding to a cumulative dose of approximately 36 to 72 µg/kg; this avoids the inter-patient dose-rate heterogeneity that limits the comparison of cumulative-dose estimates across centers. Three complementary statistical approaches were consistent with one another, although they did not identify an identical cutpoint, which supports the internal coherence of the exposure-duration signal, and the effect remained significant in the clinically most relevant subgroup of neonates with surfactant-treated respiratory distress syndrome.

Limitations should be acknowledged. The retrospective single-center design limits external generalizability and does not fully exclude residual confounding by indication: although the matched design and the adjustment for fetal distress address the strongest known confounders, neonates exposed to fentanyl may have had a baseline severity of illness that we could not fully capture. In particular, early fractional inspired oxygen and baseline ventilator settings were not systematically recorded in the study database and could therefore not be included in the models. Standardized mean differences further indicate that imbalance persisted beyond the two matched variables for several baseline characteristics, so residual confounding by baseline severity cannot be excluded. The complementary subgroup of neonates without surfactant-treated respiratory distress syndrome was too small for reliable multivariable estimation. The analysis is restricted to in-hospital respiratory outcomes; long-term respiratory and neurodevelopmental outcomes will require dedicated follow-up studies, as highlighted by our recent systematic review of neurological outcomes in mechanically ventilated neonates exposed to opioids [18]. Our findings reflect the institutional fentanyl regimen of continuous intravenous infusion at 1 µg/kg/h, which is at the lower end of the dose range described in the literature for neonatal continuous fentanyl infusion [3]. Because drug accumulation and pharmacological tolerance to fentanyl are dose-rate dependent, the exposure-duration signal observed in our cohort should not be extrapolated to higher continuous infusion rates without prospective validation; at higher rates, the temporal point beyond which respiratory harm becomes detectable may occur earlier. The decision to initiate fentanyl in the source population was at the discretion of the attending neonatologist; although all included neonates had documented moderate-to-severe pain, residual confounding by clinician’s perception of clinical severity cannot be fully excluded. Seventeen fentanyl-exposed neonates, predominantly of 22 to 26 weeks of gestation, could not be paired within the prespecified calipers and were therefore not part of the analytic cohort. Their characteristics are reported in Supplementary Table S1. This exclusion followed directly from the matching algorithm rather than from any post hoc selection: at the lowest gestational ages fentanyl-exposed neonates outnumbered unexposed ventilated neonates, so that a 1:2 pairing without replacement was frequently unattainable. Our estimates therefore apply to the matchable, predominantly moderately preterm ventilated population, and the most immature neonates are under-represented; the findings should not be extrapolated to infants born at 22 to 26 weeks, in whom fentanyl use was substantially more frequent and in whom the absence of comparable unexposed neonates precludes any inference on drug-related respiratory risk. Neonates who died before 36 weeks of postmenstrual age could not be classified for bronchopulmonary dysplasia; death is therefore a competing risk that may have influenced the estimate of the primary outcome. A sensitivity analysis using the composite outcome of bronchopulmonary dysplasia or death is reported in Supplementary Table S2. The qualifying PIPP-R score was verified in the clinical record as an eligibility criterion but was not recorded as a time-stamped variable, so the timing of the assessment could not be reported for either group. Fentanyl exposure is moreover time-varying: a longer ventilation course increases both the probability of starting fentanyl and the number of exposure days, so reverse causation and time-dependent confounding cannot be excluded.

## 5. Conclusions

Fentanyl exposure during mechanical ventilation was associated, after adjustment for gestational age and fetal distress, with a higher incidence of BPD and with a longer duration of IMV in preterm neonates. The risk of the composite respiratory outcome increased substantially when continuous fentanyl exposure extended beyond approximately 3 days. Our threshold analyses point to a possible signal around 3 days of continuous fentanyl infusion; given the observational design and the wide uncertainty of the breakpoint, this should be regarded as hypothesis-generating rather than a decision rule, and warrants prospective evaluation as a stewardship prompt. These findings support a more judicious, time-limited use of fentanyl in ventilated preterm neonates below 34 weeks of gestation: where no clear ongoing analgesic indication persists, the need for continuous infusion may reasonably be reassessed and prolonged exposure avoided. Being observational, these implications are hypothesis-generating and should inform pain- and sedation-management strategies only after confirmation in adequately powered prospective studies. Adequately powered prospective studies are needed to confirm our results.

## Figures and Tables

**Table 1:** Baseline antenatal, perinatal, and demographic characteristics of preterm neonates (<34 weeks of gestation) requiring mechanical ventilation, comparing fentanyl-exposed neonates with gestational age and birth-weight z-score matched fentanyl-unexposed neonates. Single-center cohort, NICU of Policlinico Umberto I, Sapienza University of Rome, 2015–2022. SMD, standardized mean difference; an absolute SMD > 0.10 indicates residual between-group imbalance.

Variable	Fentanyl (*n* = 50)	No Fentanyl (*n* = 92)	*p*-Value	SMD
***Maternal/obstetric characteristics***				
Maternal age > 35 years	17/47 (36.2%)	39/87 (44.8%)	0.332	−0.18
Gestational diabetes	4/50 (8.0%)	7/92 (7.6%)	1.000	0.01
Maternal thyroid disorders	8/50 (16.0%)	9/92 (9.8%)	0.276	0.19
Infectious risk	22/50 (44.0%)	36/92 (39.1%)	0.573	0.10
IUGR	6/50 (12.0%)	9/92 (9.8%)	0.681	0.07
Placental abruption	6/50 (12.0%)	14/92 (15.2%)	0.599	−0.09
Complete antenatal steroid prophylaxis	32/50 (64.0%)	64/92 (69.6%)	0.499	−0.12
Multiple pregnancy	9/50 (18.0%)	29/92 (31.5%)	0.082	−0.32
***Perinatal characteristics***				
Gestational age, weeks	28.0 (26.0–29.8)	28.0 (27.0–30.0)	0.329	−0.18
Birth weight, g	1002.5 (832.0–1260.0)	1047.5 (841.2–1347.2)	0.412	−0.13
Birth weight z-score	−0.1 (−0.9–0.5)	0.1 (−0.8–0.4)	0.717	−0.07
SGA at birth	5/50 (10.0%)	9/92 (9.8%)	1.000	0.01
Male sex	25/50 (50.0%)	56/92 (60.9%)	0.211	−0.22
Cesarean section	42/50 (84.0%)	79/92 (85.9%)	0.764	−0.05
Fetal distress ^a^	22/50 (44.0%)	20/92 (21.7%)	0.006	0.49
RDS requiring at least one dose of surfactant	44/50 (88.0%)	40/92 (43.5%)	<0.001	1.06

Notes: categorical variables are expressed as *n*/*N* (%); continuous variables as median (interquartile range). Denominators differ between rows where complete data were not available. Standardized mean differences for continuous variables were computed from group means and standard deviations; those for categorical variables from group proportions. ^a^ Fetal distress defined as umbilical cord pH below 7.2 or 5 min Apgar score below 5. Abbreviations: IUGR, intrauterine growth restriction; RDS, respiratory distress syndrome; SGA, small for gestational age. Two-sided *p* < 0.05 was considered significant.

**Table 2:** BPD and duration of IMV in the matched cohort.

Outcome	Fentanyl (*n* = 50)	No Fentanyl (*n* = 92)	*p*-Value
BPD, *n*/*N* (%)	13/50 (26.0%)	5/92 (5.4%)	<0.001
IMV duration, days, median (IQR)	4.0 (2.0–11.0)	0.0 (0.0–2.0)	<0.001

Notes: categorical data are expressed as *n*/*N* (%); continuous data as median (interquartile range). Abbreviations: BPD, bronchopulmonary dysplasia; IMV, invasive mechanical ventilation; IQR, interquartile range.

**Table 3:** Cutpoint analysis for fentanyl exposure duration and the composite respiratory outcome.

Cutpoint	*N* ≥ Cut	Events ≥ Cut	*N* < Cut	Events < Cut	Adjusted OR	95% CI	*p*-Value
≥1 d	47	21	95	10	8.79	3.02–25.61	<0.001
≥2 d	39	20	103	11	11.32	3.83–33.45	<0.001
**≥3 d**	**28**	**18**	**114**	**13**	**15.36**	**4.84–48.76**	**<0.001**
≥4 d	22	15	120	16	18.34	5.03–66.80	<0.001
≥5 d	19	14	123	17	20.32	5.19–79.49	<0.001
≥6 d	13	10	129	21	15.48	3.22–74.29	<0.001
≥7 d	11	8	131	23	8.07	1.64–39.68	0.010
≥8 d	8	5	134	26	4.72	0.83–26.77	0.080

Notes: the row in bold indicates the optimal cutpoint of 3 days; estimates for the other thresholds are exploratory. Abbreviations: CI, confidence interval; OR, odds ratio.

## Data Availability

The data presented in this study are available on request from the corresponding author due to Italian privacy legislation (Legislative Decree 196/2003); de-identified data may be released after the appropriate institutional review.

## Supplementary Materials

The following supporting information can be downloaded at: https://www.mdpi.com/article/10.3390/jcm15166457/s1, Table S1. Characteristics of the 17 fentanyl-exposed neonates who could not be matched within the prespecified calipers. Table S2. Competing-risk sensitivity analysis: composite outcome of bronchopulmonary dysplasia or death.

## Abbreviations

aOR, adjusted odds ratio; BPD, bronchopulmonary dysplasia; CI, confidence interval; GA, gestational age; IMV, invasive mechanical ventilation; IQR, interquartile range; IUGR, intrauterine growth restriction; NICU, neonatal intensive care unit; OLS, ordinary least squares; OR, odds ratio; PIPP-R, Premature Infant Pain Profile-Revised; PMA, postmenstrual age; RCS, restricted cubic spline; RDS, respiratory distress syndrome; SGA, small for gestational age; SMD, standardized mean difference; STROBE, Strengthening the Reporting of Observational Studies in Epidemiology.
