# Supplementary material for: Fentanyl Exposure Is Associated with Adverse Respiratory Outcomes in Ventilated Preterm Neonates: A Retrospective Matched Cohort Study
Source: J Clin Med. 2026 Aug 20;15(16):6457. doi: 10.3390/jcm15166457 (PMC13514582; doi:10.3390/jcm15166457)
Supplement: Supplementary file 1 [file jcm-15-06457-s001.zip › jcm-4409266-supplementary.pdf]

Supplementary Material

Table S1. Characteristics of the 17 fentanyl-exposed neonates who could not be matched within the prespecified calipers.

Characteristic	n = 17
Gestational age, weeks	24 (24–26)
Birth weight, g	640 (570–740)
Fentanyl exposure duration, days[a]	4 (2–8)
Duration of invasive mechanical ventilation, days[b]	8 (1–18)
Bronchopulmonary dysplasia at 36 weeks postmenstrual age[c]	2/12 (17%)
Death	5/17 (29%)

Data are median (interquartile range) or n/N (%). These neonates were not carried forward into the matched analytic cohort because no unexposed neonate lay within the prespecified calipers; no statistical comparison was performed.

[a] Available for 14 of the 17 neonates.

[b] Two neonates did not receive invasive mechanical ventilation and are included with a duration of zero days, as in Table 2 of the main text.

[c] Five neonates died before 36 weeks postmenstrual age and could not be assessed for this outcome.

Table S2. Competing-risk sensitivity analysis: composite outcome of bronchopulmonary dysplasia or death.

Outcome	Events	Adjusted odds ratio (95% CI)	P value
Bronchopulmonary dysplasia (primary analysis)	18	5.49 (1.72–17.54)	0.004
Bronchopulmonary dysplasia or death	32	4.72 (1.59–14.03)	0.005

Estimates are from multivariable logistic regression in the matched analytic cohort (n = 142; 50 fentanyl-exposed, 92 unexposed), adjusted for fentanyl exposure, gestational age and fetal distress. The two models are identical except for the outcome.

Fetal distress was defined as an umbilical cord pH below 7.2 or a 5-minute Apgar score below 5. CI, confidence interval.
